# Switox: Retrospective Analysis of Botulinum Toxin Switching in Management of Spasticity

**DOI:** 10.3390/toxins17030103

**Published:** 2025-02-24

**Authors:** Emilie Leblong, Patrice Piette, Carole Anne, Maud Jeanne, Marion Poyau, Anne Laure Roy, Philippe Gallien

**Affiliations:** Fondation Saint Helier, 35043 Rennes, France; patrice.piette@fondationsainthelier.com (P.P.); carole.anne@fondationsainthelier.com (C.A.); maud.jeanne@fondationsainthelier.com (M.J.); marion.poyau@fondationsainthelier.com (M.P.); annelaure.roy@fondationsainthelier.com (A.L.R.); philippe.gallien@fondationsainthelier.com (P.G.)

**Keywords:** botulinum toxin, spasticity management, biosimilar, switching

## Abstract

This retrospective study investigates botulinum toxin changes in 206 patients with spasticity, following reimbursement adjustments in France. The main objective was to evaluate the tolerance and efficacy of these changes, a topic underexplored due to the common practice of maintaining the same toxin brand. The majority of patients switched from Botox to Xeomin (73.66%), while others switched from Botox to Dysport (14.63%) or from Xeomin to Dysport (11.71%). Dose adjustments varied depending on the switch, with the change from Botox to Xeomin showing the greatest diversity in adjustments. Overall, tolerance was good, with few adverse effects reported, primarily fatigue. Perceived efficacy fluctuated, with some patients noting improvement while others experienced deterioration, but the median remained stable. A majority of patients (57.06%) chose to continue with their new treatment, indicating general satisfaction, though 42.93% preferred to return to their initial treatment. This study highlights the importance of an individualized approach and careful monitoring during toxin changes. The results suggest that toxin switches can be made without an increase in adverse effects. While differences between groups were observed, they were not statistically significant. Placebo and nocebo effects may influence perceptions of efficacy and side effects during treatment changes.

## 1. Introduction

Botulinum toxin (BoNT) is a neurotoxin whose primary site of action is nerve terminals and cranial nuclei. It reduces muscle hyperactivity by inhibiting the release of acetylcholine at the neuromuscular junction (it cleaves the 25 kDa protein associated with the synaptosome, which is necessary for vesicle docking and neurotransmitter release) [1]. As a result, BoNT injections can reduce spasticity by temporarily paralyzing muscle activity and decreasing muscle hypertonia [2]. Currently, BoNT is a first-line agent for treating focal and multifocal spasticity [3]. A large amount of research has shown that BoNT injections are effective in correcting muscle imbalances, decreasing muscle tone, and enhancing muscle function, especially in cases of hereditary spastic paraplegias (HSPs) [4], stroke [5], dystonia [6], spinal cord injury [7], cerebral palsy [8], and multiple sclerosis [9].

There are significant pharmacological differences between botulinum toxin (BoNT) preparations, particularly in terms of potency and the duration of action [10] BoNT is commercially available in France under two serotypes, A and B. Type A botulinum toxin is effective in very small amounts, with a long-lasting effect that can last for several months. These properties explain why it has been used to block neuromuscular junctions in a targeted manner through local injections [11]. It is manufactured and distributed by different laboratories (Table 1).

The units used to quantify the activity of each toxin are specific to the laboratory preparing the formulation in question. In the OnaA and AboA formulations, the neurotoxin is associated with a larger protein complex containing accessory proteins, whereas the IncoA formulation contains a purified neurotoxin, free from complexing proteins, and thus exhibits a higher specific biological activity [12]. Since each dosage is specific to the laboratory and there are no clear differences in terms of efficacy between the different preparations, their comparability remains the subject of ongoing debate [13].

IncoA was proven to be as effective as OnaA, with a comparable adverse event profile when a clinical conversion ratio of 1:1 or 1:1.2 was applied [14,15,16]. Therefore, clinical analyses demonstrate that the clinical conversion rate between OnaA and IncoA is very close to 1:1. For the conversion from OnaA to AboA, the commonly used conversion factor is ≤1:3. Studies using this conversion report clinical equivalence [17,18,19]. When the conversion factor is close to 1:3, AboA shows superior efficacy [20,21,22]. When the conversion ratio exceeds 1:3, AboA demonstrates both superior efficacy and a longer duration of action compared to OnaA, though with more adverse events [23,24,25]. While the most frequent conversion ratios are 1:3 or 1:4 [26], in clinical practice, they can vary from 1:1 [27] to 1:11 [28]. This broad spectrum of conversion ratios reflects actual clinical practice, where the treating physician decides the number of muscles to be treated and the dosage, based on the patient’s medical condition, impairment profile, and treatment objectives [13]. There is no uniform treatment strategy, and the doses used have varied significantly over the years. These total doses are not evidence-based but rather based on “expert opinion” and primarily on small clinical trials conducted by researchers [29]. Thus, there is considerable variation in clinical practices across injection sites, botulinum toxin-A brand selection, and dosing [30].

Despite the existence of dosage conversion guidelines, it is commonly accepted that these conversions should not be interchanged with other botulinum toxin preparations [31]. However, an observational study following a switch from AboA to OnaA in 48 patients demonstrated a perceived better efficacy with OnaA, with 19 adverse events reported during the AboA phase and none during the OnaA phase [32]. In another retrospective study, 87 patients who switched from AboA to ObnaA or vice versa were analyzed. No statistically significant differences in outcome measures were observed [25].

Injections are sometimes accompanied by adverse effects, with reported rates varying across studies. For children with cerebral palsy, the incidence of adverse effects ranges from 8.7% of subjects, without distinction between toxin brands [33], to as high as 23.2% [34]. However, the study by Wabbels et al., 2011 [35] found no differences in the frequency of adverse events between OnaA and IncoA, a finding supported by Saad et al., 2014 [36] or Dressler et al., 2014 [37].

Based on the available data, it cannot be conclusively stated that switching from one toxin brand to another should be avoided entirely due to loss of effectiveness or an increase in adverse effects. However, the findings suggest that such a switch can be feasible if it is carefully prepared, particularly in terms of dosage adjustments and patient-specific considerations. Proper monitoring and individualized treatment planning appear to be key factors in ensuring the switch is both effective and well-tolerated.

To address this question, an opportunity arose in France. On 1 July 2023, a publication in the Official Journal announced that only one toxin (AboA, Dysport) would continue to be reimbursed, compelling physicians to modify the toxin brand and injection patterns for their patients. Subsequently, on 25 July, a second brand (IncoA, Xeomin) was also approved for reimbursement. On 28 September 2023, the Official Journal reintroduced reimbursement for Botox toxin. Injection follow-up data are traditionally collected by the teams at the Saint-Hélier Rehabilitation department (54 rue St Helier, Rennes, France); it was determined that these data would serve as the foundation for a retrospective study.

This article adheres to the STROBE guidelines for the reporting of observational studies.

## 2. Objective

The primary objective of this study is to enhance knowledge about toxin changes and injection patterns in spastic patients following a neurological condition. The tolerance and effectiveness associated with this change will be evaluated. Such data are currently absent from the literature due to the common practice of maintaining the same toxin brands and injection patterns over time for a given patient.

## 3. Results

### 3.1. Population

Between 1 July 2023 and 28 September 2023, 230 patients who met the inclusion criteria were consulted for botulinum toxin injections and underwent a brand switch. After 28 September 2023, the patients were given the option to return to their initial type of toxin following the switch. A total of 207 patients provided consent for the use of their data in this retrospective study. For one patient, the switch was not documented, rendering the remaining data unusable. Consequently, 206 patients with consent were included in the analysis.

### 3.2. Descriptive Data

The data presented in Table 2 show the distribution of various diagnoses among the study population, with a total of 207 participants. The study population consisted of 110 females (53.14%) and 97 males (46.86%), with a mean age of 58.26 years (SD = 15.80). The most prevalent condition was stroke, which accounted for 35.27% of cases. Cerebral palsy followed as the second most common diagnosis, representing 23.67% of the participants. Multiple sclerosis was also notable, with 8.70% of the study population affected. Other conditions included spinal injuries (10.63%), dystonia (3.38%), and hereditary spastic paraplegia (2.42%). Less common diagnoses such as brain injury (2.90%), hemifacial spasm (1.45%), Parkinson’s disease (1.93%), and Guillain–Barré syndrome (0.48%) were also included. A small percentage of participants (8.21%) had other unspecified conditions. This distribution reflects the diversity of the sample and provides insight into the types of conditions treated with botulinum toxin in this cohort.

### 3.3. Main Results

#### 3.3.1. Pre-Switch Data

Table 3 presents the perceived effectiveness and satisfaction of patients prior to the switch based on the brand of botulinum toxin used (Botox vs. Xeomin), with a total of 206 participants.

For perceived effectiveness (measured in months), both Botox and Xeomin showed similar results, with a mean of 3.72 months (SD = 1.11) for Botox and 3.87 months (SD = 0.61) for Xeomin. The median for both brands was 4.00 months, with an interquartile range (IQR) of 1.00 month for Botox and a very narrow range of 0.02 months for Xeomin. The *p*-value (0.787) suggests that there was no significant difference between the two brands in terms of perceived effectiveness.

For efficacy (measured by a visual analog scale from 0 to 10), the mean score was 6.59 (SD = 2.09) for Botox and 6.95 (SD = 1.10) for Xeomin, with a median score of 7.00 for both brands and an IQR of 2.00 for Botox and 1.50 for Xeomin. The *p*-value (0.739) indicated no significant difference in efficacy between the two brands. In conclusion, the perceived effectiveness and efficacy prior to the switch showed no significant difference between Botox and Xeomin based on the statistical analysis.

Satisfaction was independent of the injecting physicians (*p* = 0.28).

#### 3.3.2. Characteristics of the Switch

The majority of switches involved Botox to Xeomin (BX), representing 73.66% of the cases. The other switches, from Botox to Dysport (BD) and Xeomin to Dysport (XD), were less frequent, comprising 14.63% and 11.71%, respectively (Table 4).

The conversion doses were calculated and standardized by two practitioners for all injecting practitioners, using a [1:3–5] ratio for Botox → Dysport and a [1:1] ratio for Botox → Xeomin. In addition to the conversion, certain dose adjustments were made based on the patient’s clinical condition and the degree of spasticity.

In the Botox-to-Dysport (BD) switch group, several dose changes were observed (Table 5). A notable case was the switch from 100 to 300 units, where a significant number of patients (5 out of 30, or 16.67%) had an increase in their dose. Additionally, a few patients (1 out of 30) experienced a more marked dosage change, such as a switch from 250 to 1000 units (3.33%). The switch to a dose of 500 units (for example, from 200 to 500) was also frequent, with four patients (13.33%) with this increase.

For the Botox-to-Xeomin (BX) switch, the dose adjustments were more varied. The change from 100 to 100 units remained the most common (21 patients out of 151, or 13.91%). Larger dose increases, such as the switch from 200 to 500 units (four patients, 23.53%) or from 250 to 500 units (one patient, 3.33%), were also observed. Overall, the dose distribution in this group appeared broader, suggesting more flexibility in dose adjustments compared to other switch types.

The Xeomin-to-Dysport (XD) group presented a smaller number of cases. The switch from 100 to 300 units was relatively rare (1 patient, 4.17%), while the switch from 200 to 500 units was much more common, with 13 patients (76.47%) in this group. Other dose adjustments, such as from 300 to 500 units or from 350 to 350 units, were also noted, but they were less frequent.

The data analysis shows that the Botox-to-Xeomin (BX) switch involved a greater variety of dose adjustments compared to the other switches. In contrast, the Botox-to-Dysport (BD) switch showed a higher concentration of patients who experienced larger dose increases, although the sample size was smaller. The Xeomin-to-Dysport (XD) switch showed fewer variations in doses, with a tendency to maintain lower or similar doses to the initial ones.

The vast majority of patients received multiple injection sites, sometimes combining sites on the upper and lower limbs. In total, 22.71% of patients had only one injection site, 27.05% had two sites, 16.91% had three sites, and 19.81% had four sites. One patient had a total of nine injection sites. It is therefore impossible to study the dependency of satisfaction based on the injected sites. The data on tolerance and satisfaction are overall and related to all the injections received during the consultation.

The injection techniques were of various types, including ultrasound, anatomical landmarks, electromyography, or stimulation. The choice of technique depended on the anatomical region and muscle activity. Thus, each site was independent of the others. Multiple detection techniques may have been used on the same patient.

We know that injection techniques can influence results [38], but no modifications in the injection technique were observed among the operators, and no specific protocol governed the switch, except for the dose equivalence calculation. However, in 80.10% of cases, the injecting physician used the same detection technique; anatomical landmarks were used in 70.25%, ultrasound in 12.3%, stimulation in 11.83%, and EMG in 5.70%. We therefore consider that the techniques and sites were comparable between the pre-switch and post-switch phases.

#### 3.3.3. Switch Assessment

A total of 206 patients underwent a brand switch in botulinum toxin, involving transitions from Botox to Dysport (BD) in 14.63% of cases, from Botox to Xeomin (BX) in 73.66% of cases, and from Xeomin to Dysport (XD) in 11.71% of cases.

Among these groups, 13 adverse events were reported at 8 weeks and finally only 8 at 4 months (Table 6), with the majority occurring in the BX group (61.54%), followed by the XD group (30.77%) and the BD group (7.69%). Fatigue was the most commonly reported adverse event, accounting for three cases, all observed in the BX group. Other reported events included leg pain, neck tingling, and urticaria on the thighs, each occurring in isolated cases and primarily within the BX and XD groups. A single case of fatigue accompanied by palpitations was noted in the BD group, representing the only case of the adverse events in this switch type. The analysis comparing adverse event rates among the three switch groups (BD, BX, and XD) using the chi-square test showed no statistically significant difference (*p* = 0.125). Although the overall occurrence of adverse events was low (7.83%), the XD group had the highest proportion of reported adverse events (18.18%), compared to 6.96% in the BX group and 3.45% in the BD group. Despite these differences in proportions, the lack of statistical significance suggests that the observed variations could be attributed to chance rather than a true effect of the specific switch type.

#### 3.3.4. Patients’ Decisions Regarding the Continuity of the Switch

After 28 September 2023, a majority of patients, 57.06%, chose to remain on the new treatment they received after the initial switch, suggesting that many found the alternative toxins, Dysport or Xeomin, satisfactory in terms of efficacy and tolerance (Table 7). However, a notable proportion of patients, 42.93%, opted to return to their original treatment when reimbursement conditions allowed, indicating that familiarity, perceived effectiveness, or individual response to the original toxin played a significant role in their decision. These findings reflect the complex interplay of factors influencing patient decisions, including perceived efficacy, tolerance, and the forced nature of the switch due to reimbursement changes. While many patients appeared to adapt well to the new toxins, a substantial number expressed a clear preference for returning to their original treatment once it became available again. This highlights the importance of individualizing treatment choices and considering patient-specific needs and experiences when managing botulinum toxin therapy.

### 3.4. Analysis

#### 3.4.1. Perceived Efficacy Evolution

**Figure 1:**
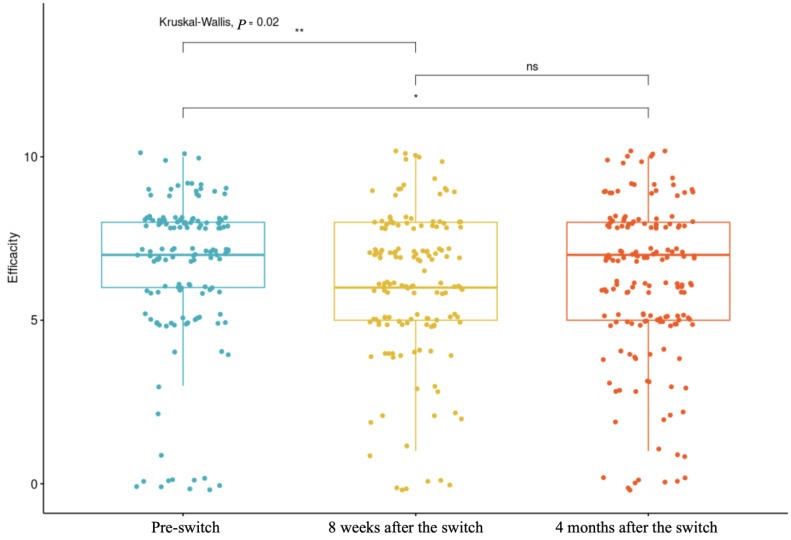
Perceived efficacy at three time points: pre-switch, 8 weeks after the switch, and 4 months after the switch.(* *p*-value < 0.1; ** *p*-value < 0.05, ns = no significant).

The boxplot presented in Figure 1 provides a visual representation of perceived efficacy at three time points: pre-switch, 8 weeks after the switch, and 4 months after the switch. The Kruskal–Wallis test showed a significant difference in perceived efficacy between the three time points (*p* = 0.02) (Table 8). It is noted that for the majority of participants, the perception remained similar, with some feeling an improvement or deterioration in the first assessment at 8 weeks. Opinions became more divided at four months, with some reporting improvements and others reporting deterioration, leading to a visually stable median, identical to the pre-switch evaluation. However, the *p*-value (at 8 weeks and 4 months, respectively; *p* adj = 0.04; *p* = 0.007) highlights a significant difference in the data, corresponding to one group feeling improved efficacy and another experiencing deterioration, both ultimately balancing around the same median.

#### 3.4.2. Predictive Factor of Efficacity Perceived

We fitted a linear model using ordinary least squares to predict adverse events at four months based on pathology, Sexe, the type of switch, tolerance, and age. The model explained a statistically significant and substantial proportion of variance (R2 = 0.63; F(5, 162) = 55.24; *p* < 0.001; adj. R^2^ =0.62). Among the predictors, pathology, Gender, the type of switch, and age showed a non-significant effect (*p* = 0.237, *p* = 0.817, *p* = 0.183, and *p* = 0.722, respectively). In contrast, tolerance demonstrated a statistically significant positive effect (*p* < 0.001). We conducted the same procedure to predict perceived efficacy. The model explained a statistically significant and weak proportion of variance (R^2^ = 0.10; F(5, 152) = 3.20; *p* = 0.009; adj. R^2^ = 0.07). As previously, only tolerance showed a statistically significant effect (*p* < 0.001). The results show a strong interrelation between perceived efficacy, tolerance, and adverse events. No other factor was identified as predictive. These data show that while tolerance was closely linked to adverse events, perceived efficacy depended on factors that were not captured in this study, likely related to pain and autonomy factors.

Moreover, no correlation was found between the injection technique and patient satisfaction at four months (r = 0.09; *p*-value = 0.32).

No significant difference was observed in the effectiveness reported by patients based on the injecting practitioners, both at 8 weeks (Kruskal–Wallis test; *p* = 0.258) and at 4 months (*p* = 0.504).

## 4. Discussion

The primary objective of this study was to enhance knowledge about toxin changes and injection patterns. The results show that even when subjected to unprepared changes, the transition is possible without worsening adverse events. However, individual clinical dimensions remain a central focus of toxin injection interventions. It is difficult to generalize these results.

### 4.1. Study Limitations and Biases

As a retrospective study, this analysis is subject to several inherent biases and limitations that must be acknowledged. The retrospective nature restricted the ability to control for confounding variables, as data collection depended on previously recorded clinical information, which may have lacked uniformity or completeness. Additionally, the absence of randomization and the reliance on real-world data made it challenging to establish causal relationships between the toxin switch and clinical outcomes. Recall bias and potential inconsistencies in follow-up duration across patients further limit the generalizability of the findings. Despite these limitations, this study provides valuable insights into the clinical practice of botulinum toxin switching, emphasizing the need for prospective, controlled studies to validate these observations.

### 4.2. Weighting of Adverse Events and Negative Perceptions

The number of individuals reporting that the new toxin was less effective than the previous one was higher than those reporting improved efficacy (32.75% vs. 20.47%). These findings must be interpreted with caution, as placebo and nocebo effects have been shown to influence outcomes when switching molecules during treatment. Studies have suggested that there is a strong placebo effect in the reduction in spasticity [5]. Similarly to placebo effects, the nocebo effect is a cognitive and idiosyncratic phenomenon with specific biological underpinnings. It is mediated by distinct neurotransmitters within well-mapped brain regions, likely located in the limbic system network. Zis et al. (2018) [39] demonstrated that adverse events related to drug treatments are widespread across various neurological disorders, particularly in conditions such as headaches, Parkinson’s disease, Alzheimer’s disease, depression, epilepsy, multiple sclerosis, and motor neuron disease. In Parkinson’s disease, it has also been shown that motor performance can be modulated in two opposing directions by placebos and nocebos, depending on positive or negative expectations regarding motor function [40]. A strong nocebo effect could negatively impact treatment adherence and overall efficacy [41].

The rates of adverse events (AEs) in placebo groups (nocebo AE rates) range from 25% in randomized controlled trials (RCTs) investigating symptomatic treatments for multiple sclerosis to nearly 80% in RCTs on motor neuron disease [42]. The grouped discontinuation rates due to AEs in placebo groups (nocebo discontinuation rates) vary from 2% in RCTs on multiple sclerosis to almost 10% in RCTs on Parkinson’s disease. In the observational study by Wetwittayakhlang et al. (2024) [43], involving patients who underwent a biosimilar switch, a nocebo effect was observed in 13.3% of cases, with a treatment discontinuation rate of 4.8%. Similarly, switching from branded medications to generics has been associated with an increase in side effects attributable to the nocebo effect [44].

During a switch, it is also possible that physicians administering the injections may have inadvertently heightened patients’ awareness of potential side effects as part of switch monitoring. However, learning about anticipated symptoms may lead to heightened self-focused attention, potentially resulting in the perception or reporting of a greater number of such symptoms [45,46]. In the context of a biosimilar switch for anti-inflammatory treatment, Tweehuysen et al. (2017) [47] reported that 24% of patients discontinued the new treatment due to an increase in subjective complaints, including a higher number of perceived painful joints and/or subjective adverse events. These outcomes are likely partially explained by nocebo effects and/or incorrect causal attributions.

However, adopting an effective communication strategy can significantly reduce the nocebo effect [42]. In an experimental setting, Varelmann et al. (2010) [48] demonstrated this during childbirth. Before administering an epidural injection, women were told one of two statements: “We are going to administer a local anesthetic that will numb the area, and you will feel comfortable during the procedure”, or “You are going to feel a big bee sting; this is the most painful part of the procedure”. Women reported significantly higher pain levels associated with the second statement.

A structured communication program during a biosimilar switch can mitigate potential nocebo effects. For instance, in the Netherlands, a “One Voice” package [49] provides health institutions with guidance on language and terminology, ensuring a standardized and unified approach to communication about biosimilar medications from the receptionist to the physician.

### 4.3. Clinical Impact of Botulinum Toxin Switching in Spasticity Management

Switching botulinum toxin formulations in the treatment of spasticity can have significant clinical implications, particularly in patients who exhibit suboptimal responses, develop tolerance, or experience adverse effects with their initial toxin. The ability to transition between different botulinum toxin products allows for personalized treatment strategies, potentially improving efficacy and patient satisfaction. Clinical observations suggest that switching may enhance therapeutic outcomes by leveraging differences in pharmacological properties, such as potency, diffusion characteristics, and immunogenicity profiles. Additionally, it provides an opportunity to optimize the treatment regimen for patients who may benefit from alternate dosing schedules or the improved targeting of specific muscle groups. However, the switch requires careful assessment of prior treatment history, dosage equivalence, and patient-specific factors to minimize risks and ensure therapeutic continuity. This retrospective analysis sheds light on the practical considerations and benefits associated with toxin switching, ultimately supporting more flexible and effective management of spasticity in diverse clinical contexts.

### 4.4. Limtation

This study has certain limitations. Firstly, the data come from only one center, so it is possible that the procedure associated with the care pathway developed by the center and used by the injecting physicians is related to the low number of adverse events. The second limitation is that the diversity of injection sites and injection guidance techniques does not allow for the identification of sites or techniques associated with lack of efficacy and satisfaction. The third limitation is that we do not have complete data on previous treatment courses and injection frequency. However, recent animal studies have shown that repeated injections lead to persistent atrophy in muscle fibers with fibrosis [50,51]. Some of the failures could be due not to the switch but to the natural progression associated with repeated injections.

## 5. Conclusions

In conclusion, this study found that transitioning between different toxin brands is feasible without increasing adverse effects, provided there is appropriate monitoring and an individualized approach. Dose adjustments varied, highlighting the importance of personalizing treatment. While the majority of patients (57.06%) chose to continue with their new treatment, a significant number (42.93%) preferred to return to their initial treatment. This suggests that factors such as habit, perceived efficacy, and individual responses to toxins play a crucial role in patient decisions. The perceived efficacy varied, with some patients experiencing improvement and others deterioration. However, the median perceived efficacy remained stable, which could be attributed to placebo and nocebo effects. Nocebo effects possibly influence the perception of efficacy and side effects during treatment changes. Despite some differences observed between the groups, these were not statistically significant. These results underline the importance of an individualized approach and careful monitoring during botulinum toxin changes. Although this study has limitations due to its retrospective nature, it provides valuable insights into real-world clinical practice and emphasizes the need for controlled prospective studies to validate these findings. In practice, physicians should consider patient preferences, adjust dosages precisely, and closely monitor potential adverse effects. Effective communication strategies should also be implemented to minimize nocebo effects and improve tolerance to treatment changes. Ultimately, this study suggests that toxin changes are possible, but their success depends on several patient-specific factors and rigorous clinical follow-up.

## 6. Materials and Methods

### 6.1. Context

The Saint-Hélier Foundation (54 rue St Helier, Rennes, France) includes, among other services, a functional rehabilitation department that treats approximately 5000 patients per year. It is also a specialized consultation center in physical medicine that routinely administers botulinum toxin injections. The switch applied to all injections administered after 1 July 2023. Reimbursement for Botox toxin was reintroduced on 28 September 2023. The protocol was subsequently written and submitted for approval. This study received a favorable opinion from the ethics committee of Rennes University Hospital on 15 November 2023. Following the approval of the ethics committee, patients who had undergone the switch and were still being followed at the Saint-Hélier rehabilitation department were contacted to obtain their consent for the collection of their data starting from the time of the switch.

### 6.2. Population

The inclusion criteria for this study required participants to be capable of providing their free and informed non-opposition to the use of their data within the framework of the study. Eligible participants were adult men or women over the age of 18 who presented with spasticity necessitating botulinum toxin injections in the upper and/or lower limbs. Exclusion criteria included individuals under legal protection measures such as guardianship, curatorship, or judicial safeguard, as well as pregnant or breastfeeding women. Additionally, patients who explicitly opposed the use of their data were not included in the study.

### 6.3. Variables and Measurements

Several criteria were analyzed using the patients’ medical records. The data collected included the patient’s pathology and information regarding the change in botulinum toxin, both before and after the switch. The doses injected prior to and following the change were also documented. Subjective evaluations by patients were assessed using a numerical scale (NS) to measure the perceived effectiveness of the previous injection on the day of reinjection, as well as their tolerance to the toxin at its peak efficacy (5 to 8 weeks post-injection) and at the time of reinjection (3 to 6 months post-injection).

Adverse effects were recorded, including their type, if present. Patients also provided subjective evaluations of the toxin’s effectiveness at peak efficacy and at the time of reinjection using the NS. Comparisons were made to assess any difference in effectiveness before and after the toxin change. Additionally, evaluations were performed using a 3-point Likert scale (worse/same/better) for the perceived effectiveness of the toxin at its peak and at the time of reinjection.

Finally, during the injection following the toxin change, the type of toxin reinjected and its total dose were documented.

### 6.4. Bias

Eight physicians specializing in physical medicine and rehabilitation (MPR) at the Pôle Saint Helier routinely administer botulinum toxin injections and thus participated in this study as injectors. The results were evaluated considering this diversity in order to eliminate any operator bias.

### 6.5. Sample

No sample size calculation was performed. The goal was to collect as many data as possible during the six-month period between the suspension and reintroduction of reimbursement. However, based on the number of patients receiving botulinum toxin injections at the Pôle Saint Helier center, it was expected that approximately 200 to 250 patient records would be analyzed over this six-month period.

### 6.6. Statistical Consideration

Statistical analyses included a description of the population based on their pathologies and a description of the type of switch with the brands and doses used. The normality of variables was assessed to determine the use of parametric or non-parametric tests. A comparison of satisfaction, tolerance, and adverse effects was conducted across the three evaluation time points: the first evaluated injection efficacy before the switch, the second evaluated efficacy approximately 8 weeks after the switch, and the third evaluated efficacy approximately 4 months after the switch. Predictive factors of satisfaction were analyzed using multivariate analyses. No data imputation was performed for missing data. The statistics were conducted using RStudio v. 2024.12.1-563.

## Figures and Tables

**Table 1:** Botulinum neurotoxin preparations and manufacturers.

BoNT Preparation	Brand Name (Manufacturer)
OnabotulinumtoxinA (OnaA)	Botox (Allergan, Inc., Irvine, CA, USA)
AbobotulinumtoxinA (AboA)	Dysport (Ipsen Ltd., Paris, France)
IncobotulinumtoxinA (IncoA)	Xeomin (Merz Pharmaceuticals, Frankfurt, Germany)

**Table 2:** Characteristics and distribution of diagnoses among study population (N = 207).

Disease	Overall
Stroke	73 (35.27%)
Cerebral palsy	49 (23.67%)
Spinal injuries	22 (10.63%)
Multiple sclerosis	18 (8.70%)
Dystonia	7 (3.38%)
Brain injury	6 (2.90%)
Hereditary spastic paraplegia	5 (2.42%)
Parkinson’s disease	4 (1.93%)
Hemifacial spasm	3 (1.45%)
Brain tumor	1 (0.48%)
Encephalopathy	1 (0.48%)
Guillain–Barré syndrome	1 (0.48%)
Others	17 (8.21%)
Female = 110 (53.14%)	Male = 97 (46.86%)
Age: mean (SD)	58.26 (15.80)

SD = standard deviation.

**Table 3:** Perceived effectiveness and satisfaction prior to switch by toxin brand.

	Botox	Xeomin	Overall	
	n = 182	n = 24	n = 206	*p*-Value (Wilcox)
*Perceived effectiveness (in months)*				
Mean (SD)	3.72 (1.11)	3.87 (0.61)	3.74 (1.06)	0.787
Median (IQR)	4.00 (1.00)	4.00 (0.02)	4.00 (1.00)	
*Efficacity (VAS from 0 to 10)*				
Mean (SD)	6.59 (2.09)	6.95 (1.10)	6.63 (2.00)	0.739
Median (IQR)	7.00 (2.00)	7.00 (1.50)	7.00 (2.00)	

SD = standard deviation; IQR = interquartile rank.

**Table 4:** Table of switches.

Switch	n = 206
Botox → Dysport (BD switch)	30 (14.63%)
Botox → Xeomin (BX switch)	151 (73.66%)
Xeomin → Dysport (XD switch)	24 (11.71%)

**Table 5:** Dose equivalence by switch type.

Switch Dose	BD	BX	XD
Count (%)	30 (14.63%)	151 (73.66%)	24 (11.71%)
(Row, %) (Col, %)			
100 → 100	-	21 (100.00%) (13.91%)	-
100 → 150	-	2 (100.00%) (1.32%)	-
100 → 300	5 (83.33%) (16.67%)	-	1 (16.67%) (4.17%)
100 → 500	-	-	1 (100.00%) (4.17%)
150 → 100	-	1 (100.00%) (0.66%)	-
150 → 150	-	11 (100.00%) (7.28%)	-
150 → 300	2 (100.00%) (6.67%)	-	-
150 → 400	2 (100.00%) (6.67%)	-	-
150 → 500	3 (100.00%) (10.00%)	-	-
190 → 500	-	-	1 (100.00%) (4.17%)
20 → 20	-	1 (100.00%) (0.66%)	-
200 → 100	-	1 (100.00%) (0.66%)	-
200 → 200	-	26 (100.00%) (17.22%)	-
200 → 250	-	2 (100.00%) (1.32%)	-
200 → 300	-	2 (100.00%) (1.32%)	-
200 → 400	-	1 (100.00%) (0.66%)	-
200 → 500	4 (23.53%) (13.33%)	-	13 (76.47%) (54.17%)
200 → 600	1 (50.00%) (3.33%)	-	1 (50.00%) (4.17%)
200 → 800	1 (50.00%) (3.33%)	-	1 (50.00%) (4.17%)
250 → 1000	1 (100.00%) (3.33%)	-	-
250 → 200	-	1 (100.00%) (0.66%)	-
250 → 250	-	14 (100.00%) (9.27%)	-
250 → 300	-	2 (100.00%) (1.32%)	-
250 → 400		1 (100.00%) (3.33%)	-
250 → 600	1 (100.00%) (3.33%)	-	-
250 → 800	1 (100.00%) (3.33%)	-	-
27.5 → 27.5	-	1 (100.00%) (0.66%)	-
300 → 300	-	13 (100.00%) (8.61%)	-
300 → 500	1 (50.00%) (3.33%)	1 (50.00%) (0.66%)	-
300 → 800	-	-	1 (100.00%) (4.17%)
350 → 350	-	8 (100.00%) (5.30%)	-
350 → 500		-	1 (100.00%) (0.66%)
350 → 800	1 (100.00%) (3.33%)	-	-
350 → 950	1 (100.00%) (3.33%)	-	-
400 → 1000	2 (66.67%) (6.67%)	-	1 (33.33%) (4.17%)
400 → 200	-	1 (100.00%) (0.66%)	-
400 → 400	-	14 (100.00%) (9.27%)	-
400 → 500	1 (50.00%) (3.33%)	1 (50.00%) (0.66%)	-
400 → 800	1 (50.00%) (3.33%)	-	1 (50.00%) (4.17%)
450 → 450	-	6 (100.00%) (3.97%)	-
450 → 500	-	1 (100.00%) (0.66%)	-
50 → 100	-	1 (100.00%) (0.66%)	-
50 → 300	-	-	1 (100.00%) (4.17%)
50 → 50	-	6 (100.00%) (3.97%)	-
50 → 75	-	1 (100.00%) (0.66%)	-
500 → 1150	-	-	1 (100.00%) (4.17%)
500 → 400	-	1 (100.00%) (0.66%)	-
500 → 500	-	7 (100.00%) (4.64%)	-
550 → 550	-	1 (100.00%) (0.66%)	-
600 → 1500	1 (50.00%) (3.33%)	-	1 (50.00%) (4.17%)
600 → 600	-	1 (100.00%) (0.66%)	-
65 → 65	-	1 (100.00%) (0.66%)	-

BD = Botox → Dysport; BX = Botox → Xeomin; XD = Xeomin → Dysport.

**Table 6:** Assessment of toxin injection post-switch.

	BD	BX	XD	Overall	*p*-Value
**Eight-week point**					
*Tolerability (VAS from 0 to 10)*					Kruskal–Wallis
Mean (SD)	9.04 (1.90)	9.57 (1.35)	8.86 (2.05)	9.39 (1.58)	0.07 *
Median (IQR)	10.00 (0.25)	10.00 (0.00)	10.00 (1.50)	10.00 (0.00)	
Missing	2	36	2	40	
*Efficacity (VAS from 0 to 10)*					
Mean (SD)	5.77 (2.10)	6.16 (2.43)	6.25 (1.83)	6.11 (2.30)	0.63
Median (IQR)	6.00 (3.25)	7.00 (3.00)	6.00 (3.00)	6.25 (3.00)	
Missing	6	51	4	61	
*Perceived difference*					Chisq square
No	21 (72.41%)	59 (52.68%)	13 (59.09%)	93 (57.06%)	0.16
Yes	8 (27.59%)	53 (47.32%)	9 (40.91%)	70 (42.94%)	
Missing	1	39	2	42	
*Quality of difference*					Chisq square
Better	4 (13.79%)	18 (15.65%)	2 (9.09%)	24 (14.46%)	0.32
Same	21 (72.41%)	61 (53.04%)	13 (59.09%)	95 (57.23%)	
Worse	4 (13.79%)	36 (31.30%)	7 (31.82%)	47 (28.31%)	
Missing	1	36	2	39	
*Adverse event*					Chisq square
No	28 (96.55%)	107 (93.04%)	18 (81.82%)	153 (92.17%)	0.12
Yes	1 (3.45%)	8 (6.96%)	4 (18.18%)	13 (7.83%)	
Missing	1	36	2	39	
**Four-month point**					
*Tolerability (VAS from 0 to 10)*					Kruskal–Wallis
Mean (SD)	9.35 (2.12)	9.45 (1.37)	9.36 (1.56)	9.43 (1.51)	0.988
Median (IQR)	10.00 (0.00)	10.00 (0.00)	10.00 (0.00)	10.00 (0.00)	
Missing	7	29	2	38	
*Efficacity (VAS from 0 to 10)*					
Mean (SD)	6.07 (2.56)	6.13 (2.37)	6.40 (2.87)	6.15 (2.45)	0.672
Median (IQR)	6.00 (2.75)	7.00 (3.00)	7.50 (3.25)	7.00 (3.00)	
Missing	8	34	4	46	
*Perceived difference*					Chisq square
No	11 (47.83%)	63(50.00%)	8 (38.10%)	82 (48.24%)	0.60
Yes	12 (52.17%)	63 (50.00%)	13 (61.90%)	88 (51.76%)	
Missing	7	25	3	35	
*Quality of difference*					0.29
Better	5 (21.74%)	22 (17.32%)	8 (38.10%)	35 (20.47%)	
Same	11(47.83%)	61 (48.03%)	8 (38.10%)	80 (46.78%)	
Worse	7 (30.43%)	44 (34.65%)	5 (23.81%)	56 (32.75%)	
Missing	7	24	3	34	
*Adverse event*					0.58
No	23 (95.83%)	119 (95.97%)	20 (90.91%)	162 (95.29%)	
Yes	1 (4.17%)	5 (4.03%)	2 (9.09%)	8 (4.71%)	
Missing	6	27	2	35	

* *p*-value < 0.1. VAS = visual analogique scale; SD = standard deviation; IQR = interquartile rank; BD = Botox → Dysport; BX = Botox → Xeomin; XD = Xeomin → Dysport.

**Table 7:** Distribution of botulinum toxin switches and patient preferences.

Type Evolution	n = 177	Keep New	Back to Original
Botox–Dysport–Botox	9 (5.08%)	no	yes (5.08%)
Botox–Dysport–Dysport	16 (9.04%)	yes (9.04%)	no
Botox–Xeomin–Botox	60 (33.90%)	no	yes (33.90%)
Botox–Xeomin–Xeomin	70 (39.55%)	yes (39.55%)	no
Xeomin–Dysport–Dysport	15 (8.47%)	yes (8.47%)	no
Xeomin–Dysport–Xeomin	7 (3.95%)	no	yes (3.95%)
		57.06%	42.93%

**Table 8:** Comparative analysis of perceived efficacy at different time points.

*Efficacity (VAS from 0 to 10)*						
Group 1	Group 2	*p*	*p*.adj	*p*.format	*p*.signif	Method
Pre-switch	8 weeks after switch	0.0228	0.046	0.0228	*	Wilcoxon paired
Pre-switch	4 months after switch	0.00244	0.007	0.0024	**	Wilcoxon paired
8 weeks after switch	4 months after switch	0.733	0.73	0.7335	ns	Wilcoxon paired
Overall		0.02			*	Kruskall–Wallis
*Quality of difference*						
	8 weeks after switch	4 months after switch				
				0.132	ns	Chisq.2
Better	24 (14.46%)	35 (20.47%)				
Same	95 (57.23%)	80 (46.78%)				
Worse	47 (28.31%)	56 (32.75%)				

* *p*-value < 0.1; ** *p*-value < 0.05; ns = no significant.

## Data Availability

The data presented in this study, due to privacy are not publicly available but data are available on request from the corresponding author.

## Abbreviations

The following abbreviations are used in this manuscript:

MDPI	Multidisciplinary Digital Publishing Institute
DOAJ	Directory of Open Access Journals
BoNT	Botilinum Neuro Toxine
OnaA	OnabotulinumtoxinA
AboA	AbobotulinumtoxinA
IncoA	IncobotulinumtoxinA
BD	Botox → Dysport switch
BX	Botox → Xeomin switch
XD	Xeomin → Dysport switch
